# Engagement, Satisfaction, and Therapeutic Alliance With an AI Conversational Agent for Parents: Quantitative Descriptive Study

**DOI:** 10.2196/100302

**Published:** 2026-08-19

**Authors:** Karin Mostovoy, Blanca S Pineda, Arjun Bharat, Tyrique Patterson, Jing Mao, Carlos Felipe Rivera-Cepeda, Daniel M Bagner, Antonio Y Hardan, Eduardo L Bunge

**Affiliations:** 1 Department of Psychology, Palo Alto University, 1791 Arastradero Rd, Palo Alto, CA, 94304, United States, 1 (800) 818-6136; 2 Institute for International Internet Interventions for Health (i4Health), Palo Alto University, Palo Alto, CA, United States; 3 Department of Psychology, Universidad Santo Tomás, Temuco, Chile; 4 Center for Children and Families, Department of Psychology, Florida International University, Miami, FL, United States; 5 Department of Behavioral Sciences, Stanford University Medical Center, Stanford University, Stanford, CA, United States; 6 ParenteAI, Mountain View, CA, United States; 7 Children and Adolescent Psychotherapy and Technology Research Lab, Palo Alto University, Palo Alto, CA, United States

**Keywords:** artificial intelligence, AI, conversational agents, parent management training, therapeutic alliance., digital mental health

## Abstract

**Background:**

Parent management training (PMT) is an evidence-based intervention for addressing child behavioral difficulties; however, caregivers often need additional guidance when implementing skills in daily life. Pat is an AI conversational agent designed to augment a therapist-led PMT program by providing caregivers with real-time guidance, reinforcement, and answers to parenting questions between sessions.

**Objective:**

This study explored caregivers’ engagement with Pat, satisfaction, and the therapeutic alliance formed between caregivers and Pat during online group PMT.

**Methods:**

Data from 88 caregivers of children aged 3 to 14 years participating in an online PMT program were analyzed at 3 time points (wk 4, 8, and 12). Engagement with Pat was measured through the number of messages exchanged with Pat, modules completed, in-the-moment support chats, and days the platform was used. Satisfaction with the intervention was measured using the Net Promoter Score and the Therapy Attitude Inventory. The therapeutic alliance between caregivers and Pat was assessed using the Working Alliance Inventory–Short Form Revised.

**Results:**

Caregivers exchanged a high number of messages with Pat (mean 383.09, SD 185.64 by wk 4 and mean 726.47, SD 405.41 by wk 8) and completed multiple required and optional modules (mean 5.41, SD 1.93 at wk 4; mean 10.49, SD 9.18 at wk 8). Net Promoter Scores remained high and stable across the study (wk 4=77.8, wk 8=76.7, and wk 12=75.0), with no significant change over time (*F*_2,114_=0.10; *P*=.91). Therapy attitudes increased significantly from week 4 (mean 4.22, SD 0.47) to week 8 (mean 4.49, SD 0.37) and remained stable through week 12 (mean 4.49, SD 0.48, *F*_2, 114_=5.69; *P*=.004). Therapeutic alliance ratings with Pat were consistently high across time points (mean 4.10, SD 0.63 at wk 4; mean 4.10, SD 0.55 at wk 8; and mean 4.04, SD 0.63 at wk 12), with no significant differences across weeks (*F*_2, 114_=0.182; *P*=.83).

**Conclusions:**

These findings show that caregivers meaningfully engaged with Pat, remained highly satisfied across time, established a strong therapeutic alliance, and held positive therapy attitudes. Overall, the hybrid group model integrating human therapists with Pat may represent a promising and efficient strategy for enhancing engagement and providing scalable and real-time support to parents.

## Introduction

Group parent management training (PMT) programs have been shown to effectively reduce children’s behavioral problems [1,2] and improve parenting practices and caregiver mental health [1,3]. These interventions have also been associated with reductions in harsh parenting behaviors and improvements in children’s social functioning, including school-based outcomes [4,5]. Importantly, PMT remained effective even when children received additional services, supporting consistent gains in behavior and parenting skills [3]. Despite this strong evidence, access to behavioral parenting interventions remains a significant challenge. Many families do not receive evidence-based care due to barriers such as time constraints, transportation challenges, limited service availability, and lack of childcare [6,7]. These challenges highlight the need for more accessible, flexible, and scalable approaches to delivering parenting support in real-world settings.

Emerging evidence suggests that integrating digital tools into parenting interventions may enhance treatment effectiveness and accessibility. Technology-enhanced PMT programs have demonstrated improved outcomes in some contexts, including greater reductions in child behavior intensity for certain parent profiles [8]. Adjunctive digital tools, such as mobile apps, have also been associated with increased treatment efficiency, including faster mastery of parenting skills, improved homework completion, and increased participation between sessions without reductions in satisfaction [9,10]. Digital enhancements may further support the maintenance of treatment gains, with evidence of improved parenting practices and reduced disruptive child behavior at follow-up [11]. AI-based tools may offer additional advantages by providing real-time, personalized support outside scheduled sessions, which helps reduce barriers [12,13]. Early research on AI-driven parenting conversational agents (CAs) has shown promising results, including high completion, retention, and user satisfaction rates, as well as a strong likelihood of recommendation, which supports their potential to enhance engagement and scalability [14-16]. Entenberg et al [16] highlighted that even brief digital interventions delivered via a CA can promote caregiver learning and increase accessibility to parenting strategies. Entenberg et al [16] found that caregivers exchanged an average of 49.8 (SD 1.53) messages with a CA, highlighting the feasibility of sustained interaction even with earlier forms of the technology.

The recent emergence of generative AI has expanded the capabilities of CAs. To our knowledge, the only published studies examining a generative AI agent in the context of PMT have used Parente AI, a therapist-facing platform that includes an AI agent, called Pat, that supports parents between sessions. Pat was initially tested in simulations in which the treatment fidelity was compared with that of a human therapist [17]. Regarding safety and fidelity, emerging evidence indicates that Pat can deliver parenting content in accordance with established treatment protocols [17]. Desage et al [17] reported preliminary findings demonstrating high treatment fidelity scores when CAs were used to distribute PMT content, supporting their potential to reliably deliver evidence-based material. Vaclavik et al (unpublished data, 2025) conducted a case series combining Pat with weekly therapist phone calls, in which Pat demonstrated high treatment fidelity, did not include any inappropriate content, and referred users to consult a mental health professional when necessary, which supports the safe and responsible use of the CA.

Rivera-Cepeda et al [18] conducted a feasibility study combining 4 sessions of a human therapist plus Pat and reported significant reductions in both externalizing and internalizing behaviors within a quasi-experimental design, alongside significant improvements in parental depression, anxiety, and stress and the development of a strong therapeutic alliance. Rivera-Cepeda et al [18] demonstrated that caregivers exchanged an average of 376 messages, suggesting that AI training and responsiveness improved from previous technologies (see Entenberg et al [16]) and may facilitate deeper, more sustained user interaction. Together, these findings indicate that CAs can support caregiver engagement, particularly as the technology continues to evolve. Although prior studies have examined the feasibility and preliminary clinical outcomes associated with Pat, less is known about how caregivers engage with Pat over time, how satisfaction evolves throughout treatment, and how it is maintained with an AI CA within a hybrid PMT program.

More recently, research has begun to explore the integration of AI in group-based parenting interventions. A prior qualitative study examining the combination of group PMT and an AI CA found that caregivers reported valuing the additional support provided between sessions, particularly the ability to access guidance in real time when facing parenting challenges [19]. Caregivers also described the AI agent as a helpful supplement to therapist-led sessions, enhancing their ability to apply skills in daily life. These findings suggest that integrating AI into group PMT may offer unique benefits, such as 24/7 personalized access to guidance; however, quantitative data examining engagement, satisfaction, and therapeutic processes within this combined format remain limited.

Although group-based PMT programs are effective, caregivers may require more individualized and immediate support than group formats alone can provide. Integrating AI CAs offers a potential solution by delivering personalized guidance between sessions. While prior research has demonstrated promising outcomes, most studies have focused on one-to-one formats and quantitative measures, with limited attention to caregiver experiences in hybrid models. Furthermore, in Rivera-Cepeda et al [19], caregivers reported highly positive experiences with both therapist-led sessions and Pat, an AI CA, highlighting the value of group support alongside Pat’s structure, practical tools, and constant availability. Notably, many caregivers attributed a greater portion of their progress to Pat; however, this appears to be driven by the immediacy of its in-the-moment support, which enabled caregivers to apply strategies directly in real-life situations. These findings suggest that AI-supported PMT may extend the impact of therapy by providing continuous, context-sensitive support that complements human-delivered care.

Thus, whereas prior studies involving Pat have focused on feasibility, preliminary clinical outcomes, or qualitative caregiver experiences, this study examines quantitative indicators of caregiver engagement, satisfaction, and therapeutic alliance across multiple time points within a hybrid group PMT program. Despite growing evidence supporting the use of AI-based CAs in parenting interventions, several important gaps remain. In particular, little is known about caregiver engagement when AI tools are integrated into group-based PMT formats. Additionally, there is a limited understanding of how caregivers perceive the treatment and the therapeutic alliance with Pat across different stages of treatment. Addressing these gaps is critical for evaluating the feasibility, acceptability, and potential clinical utility of integrating AI into existing evidence-based parenting programs. Building on prior studies examining feasibility and preliminary outcomes, this study aims to examine caregivers’ engagement with the AI CA, Pat; to assess satisfaction across multiple time points; and to evaluate the therapeutic alliance formed between caregivers and the AI agent during participation in an online group-based PMT program.

## Methods

### Study Design

This study is a secondary analysis of data collected during a real-world implementation of a group PMT program enhanced by an AI CA called “Pat.” The dataset has previously been examined in a separate secondary analysis focusing on qualitative parent experiences [19]. The current manuscript analyzes the quantitative components of the same implementation dataset, examining indicators of feasibility and acceptability, including parent engagement, satisfaction, and perceived therapeutic alliance with Pat.

### Participants

As previously described in Rivera-Cepeda et al [19], the participants consisted of 88 primary caregivers, the majority of whom were female (n=75, 86%). Caregivers ranged in age from 34 to 65 years (mean 44.31, SD 5.73) and had a child aged between 3 and 14 years (mean 7.98, SD 2.45). Most children were male (n=70, 80%), while 20% (n=18) were female. Of the 88 caregivers, 74 (84%) identified as White or of European descent, 9 (10%) identified as another race or ethnicity, and 3 (3%) identified as Indigenous or Native people. An additional 3 (3%) caregivers did not report their race or ethnicity, as this information was optional. In terms of relationship status, over half of caregivers were married (n=49, 56%), 17% (n=15) were living with a partner, 9.2% (n=8) were single, 9.2% (n=8) were divorced, and 8.0% (n=7) were separated. Educational attainment was generally high: 42% (n=37) had completed a postgraduate degree (eg, specialization, master’s, or doctorate), 38% (n=33) held a university degree, 15% (n=13) completed technical or nonuniversity training, 4.5% (n=4) completed 12 years of schooling, and 1.1% (n=1) reported fewer than 7 years of formal education [19].

### Recruitment

Caregivers in Argentina and Paraguay were recruited through online outreach conducted by licensed mental health professionals in each country. Recruitment occurred on a rolling basis from January 2025 to November 2025 and targeted caregivers of children aged 3 to 12 years with disruptive behavior concerns. Interested caregivers completed online registration and were enrolled in the ParenteAI program.

As described in Rivera-Cepeda et al [19], the program included access to the ParenteAI digital platform and participation in a live, therapist-led parenting group delivered via videoconference. Study measures were administered at midintervention (wk 4) and postintervention (wk 8 or wk 12).

### Parent Management Training Hybrid Model: Combining a Human Therapist and AI

The intervention followed the hybrid PMT model integrating therapist-led group sessions with the ParenteAI chatbot (“Pat”), as detailed in Rivera-Cepeda et al [19]. Caregivers attended live sessions and engaged with Pat between meetings to support skill acquisition and implementation. Live sessions focused on core PMT principles (eg, reinforcement strategies, effective instruction, praise, and consequences), while Pat provided structured modules and personalized in-the-moment support. Caregivers interacted with Pat through conversational exchanges that included psychoeducation, skill practice, role-play activities, personalized reminders, responses to parenting questions, and support for applying PMT strategies to real-life situations between sessions. Therapists reviewed caregiver engagement with Pat, provided feedback, and assigned weekly modules.

Cohorts 1 and 2 were delivered over a 12-week period and included 8 required modules. Subsequent cohorts were condensed to an 8-week format. While the number of required modules remained consistent across all cohorts, later cohorts were offered 9 additional optional PMT modules reflecting iterative refinements to Pat. Module completion was scheduled according to consistent group timelines across cohorts. Although the core intervention content remained consistent, changes in program duration and the availability of optional modules led to a more condensed delivery and greater opportunities for additional engagement.

### Measures

#### Engagement

Participant engagement with the digital platform was assessed using objective usage metrics captured within the ParenteAI app. Engagement indicators included the number of modules completed, the number of in-the-moment support conversations initiated with Pat, the total number of messages exchanged between caregivers and Pat, the average number of messages sent by caregivers per conversation, and the total number of words sent by caregivers across all interactions. Engagement was also measured by the number of modules completed and the number of Q&As initiated by caregivers. The modules are structured, required learning units designed for caregivers to complete in order to ensure exposure to the core therapeutic components. The Q&As are an optional component of the program designed to provide caregivers with in-the-moment support, as well as problem-solving with questions that they may have about the interventions or modules.

#### Net Promoter Score

Caregivers were asked to rate their willingness to recommend the intervention on a Likert scale ranging from 1 (“strongly disagree”) to 10 (“strongly agree”). Scores are categorized as promoters (9-10), passives (7-8), and detractors (0‐6). After providing the score, users were asked, “Why did they provide that score?” The Net Promoter Score (NPS) is calculated by subtracting the percentage of detractors from the percentage of promoters, resulting in a score between −100 and 100. The NPS has been proposed as a measure to evaluate the overall impression of a digital product and has been used in other CA studies [20]. General guidelines for interpreting the score show that negative scores (below 0) indicate more detractors than promoters; a score from 0 to 30 is considered “good,” 30 to 70 is considered “great,” and 70 to 100 is the “excellent” or “world-class” tier.

#### Working Alliance Inventory

The Working Alliance Inventory (WAI) is a well-established measure that is used to assess therapeutic alliance through a total score as well as 3 subscale scores reflecting bond, goal, and task. To assess the therapeutic alliance, the Working Alliance Inventory–Short Revised (WAI-SR) was used. The WAI-SR is a shortened and refined version that was developed to efficiently capture the core components of the alliance. Prior research has demonstrated that the WAI-SR has strong psychometric properties, including high internal consistency (Cronbach α>0.80) and good convergent validity, particularly with the Helping Alliance Questionnaire, with correlations exceeding 0.64 (*P*< .001). Confirmatory factor analysis supports the 3-factor structure of the WAI-SR, with acceptable to good model fit observed across treatment findings, suggesting that the WAI-SR consistently assesses the same underlying constructs and is appropriate for use across diverse psychotherapy contexts [21].

#### Therapy Attitude Inventory

The Therapy Attitude Inventory (TAI) was used to assess caregiver satisfaction with PMT, parent-child treatments, and family-based therapy. The measure consists of 10 items rated on a 5-point Likert scale, with response options ranging from 1, reflecting dissatisfaction or perceived worsening of problems, to 5, reflecting high satisfaction or perceived improvement. Total scores ranged from 10 to 50, with higher scores indicating greater satisfaction with treatment. A Spanish version of the TAI was examined by Neuman et al [22], who were the first to evaluate its psychometric properties in Latinx populations in the United States. This version demonstrated excellent internal consistency (Cronbach α=0.92) and strong test-retest reliability over a 1-week interval (*r*=0.86). Evidence of construct validity has been supported by a strong positive association with the WAI-SR (*r*=0.67) and a moderate negative association with the Strengths and Difficulties Questionnaire (*r*=–0.46; Neuman et al [22]). Building on this validated version, a committee of experts adapted the Spanish version of the TAI, which was used in this study.

### Ethical Considerations

As described in Rivera-Cepeda et al [19], the University of Chile Institutional Review Board approved this study (approval number: 23136643/2023). This study was a secondary data analysis of deidentified data. Caregivers consented to the use of anonymized data at enrollment through the ParenteAI Terms of Service. Further consent was obtained when participants completed the surveys as part of their participation in the hybrid group PMT. Participants could leave the study at any time.

### Data Analysis

Engagement, NPS, WAI, and TAI descriptives were reported using descriptive analysis. Descriptive statistics were calculated to characterize user engagement across weeks 4, 8, and 12, including total messages exchanged, Q&A interactions, modules completed, and days of platform use. Additionally, ANOVAs were run for NPS, WAI, and TAI. For the NPS, there were a total of 117 observations analyzed, corresponding to 88 unique participants. Of these, 59 participants contributed 1 measurement and 29 contributed 2 measurements; no participant contributed more than 2 measurements. NPS categories (promoters, passives, and detractors) were calculated at the observation level, using all scores provided rather than aggregating by participant. A linear mixed-effects model was used to examine differences in NPS across weeks 4, 8, and 12, accounting for partially repeated measurements. Measurement week was included as a fixed effect, and participant identifier was included as a random intercept. The linear mixed-effects model was selected because some participants contributed data at multiple time points, resulting in partially repeated measurements. This approach retained all available NPS scores while accounting for within-participant clustering. To facilitate interpretation and comparison across data points, descriptive statistics and exploratory ANOVAs were also conducted. Although some participants contributed data at multiple time points, week was treated as a between-subjects factor for these exploratory analyses due to the limited overlap of participants across data points.

## Results

### Engagement

#### Messages Exchanged

A total of 88 caregivers were included in the quantitative analyses. Participant demographic characteristics are presented in Table 1. By week 4, participants exchanged an average of 383.09 (SD 185.64; n=54) messages, and by week 8, they exchanged an average of mean 726.47 (SD 405.41; n=43) messages. The first 2 parenting cohorts had fewer modules available and did a 12-week program, exchanging an average of 605.00 (SD 268.61; n=20) messages.

**Table 1.** Engagement descriptives.

Measure	Overall mean (SD)	Median (IQR)	Week 4 (n=54), mean (SD)	Week 8 (n=43), mean (SD)	Week 12 (n=20), mean (SD)
Messages exchanged	547.22 (334.81)	447 (107‐1855)	383.09 (185.64)	726.47 (405.41)	605.00 (268.61)
Modules completed	7.00 (3.05)	7 (1‐19)	5.41 (1.93)	8.67 (3.39)	7.70 (2.66)
Days used	14.25 (8.60)	11 (2‐42)	10.00 (5.15)	17.42 (9.26)	18.90 (9.53)
Q&A interactions	8.08 (7.94)	6 (0‐40)	5.17 (4.75)	10.49 (9.18)	10.75 (9.51)

#### Modules Completed and Days Used

When examined by week, module completion increased from week 4 (mean 5.41, SD 1.93; n=54) to week 8 (mean 8.67, SD 3.39; n=43) and remained relatively high at week 12 (mean 7.70, SD 2.66; n=20). Similarly, days of platform use increased from week 4 (mean 10.00, SD 5.15) to week 8 (mean 17.42, SD 9.26) and week 12 (mean 18.90, SD 9.53), suggesting sustained engagement among participants who remained active at later sessions.

#### Q&A Interactions

Although Q&A interactions were optional, mean Q&A interactions increased at week 4 (mean 5.17, SD 4.75; n=54) and week 8 (mean 10.49, SD 9.18; n=43) and remained similar at week 12 (mean 10.75, SD 9.51; n=20). Similar to message exchange, variability increased at later time points.

### Satisfaction: NPS

Rivera-Cepeda et al [19] reported an excellent NPS score at the end of the treatment (76.92) but did not report the NPS across weeks. For week 4, based on 54 respondents, the NPS was 77.7. For week 8, based on 43 respondents, the NPS was 76.8, and for week 12, based on 20 respondents, the NPS was 75. Results from the linear mixed-effects model indicated no significant effect of week on NPS (*F*_2, 38.67_=1.66; *P*=.20). Estimated marginal means of NPS remained high across all assessment points (wk 4: mean 9.13, 95% CI 8.81-9.46; week 8: mean 9.34, 95% CI 9.00-9.68; and wk 12: mean 9.43, 95% CI 8.96-9.91), showing a descriptive upward trend, although differences between time points were not statistically significant. Descriptive statistics indicated consistently high NPS ratings across all time points. Mean NPS scores were 9.33 (SD 1.13; n=54) at week 4, 9.35 (SD 1.04; n=43) at week 8, and 9.20 (SD 2.12; n=20) at week 12. The overall mean NPS across the sample was 9.32 (SD 1.31; n=117), with scores ranging from 2 to 10 (Table 2). The Levene test for equality of variances was nonsignificant (*F*_2, 114_=2.59; *P*=.09) indicating that the assumption of homogeneity of variance was met. The overall ANOVA was not significant (*F*_2, 114_=0.10; *P*=.90; *η*²=0.002) suggesting that NPS did not differ across time points. The week accounted for less than 1% of the variance in NPS scores. CIs around the effect size estimate were small and included zero, further supporting the absence of meaningful differences over time. These findings indicate that participants reported consistently high levels of likelihood to recommend the program across weeks 4, 8, and 12, with no evidence of change over time.

**Table 2.** Net Promoter Score (NPS) descriptives and estimated marginal means by week^a^.

Week	Participants, n	Promoters, n (%)	Passives, n (%)	Detractors, n (%)	NPS	Estimated mean (SE; 95% CI)	
4	54	43 (79.6)	10 (18.5)	1 (1.9)	77.8	9.13 (0.16; 8.81-9.46)	
8	43	34 (79.1)	8 (18.6)	1 (2.3)	76.7	9.34 (0.17; 9.00-9.68)	
12	20	17 (85)	1 (5)	2 (10)	75	9.43 (0.24; 8.96-9.91)	

a Note promoters scored 9-10, passives 7-8, and detractors 0-6. NPS % promoters − % detractors. Estimated marginal means from a linear mixed-effects model with random intercepts for participants.

### TAI

Mean scores for TAI across weeks were week 4 (mean 4.22, SD 0.47), week 8 (mean 4.49, SD 0.37), and week 12 (mean 4.49, SD 0.48). Regarding the ANOVA comparing TAI scores across weeks, although some participants contributed data at multiple time points, week was treated as a between-subjects factor for these exploratory analyses. The Levene test was nonsignificant *(F*_2, 114_=0.80; *P*=.45) indicating that the assumption of homogeneity of variance was met. The overall ANOVA was significant (*F*_2, 114_=5.69; *P*=.004; *η*^2^_p_=0.09) suggesting that TAI scores differed across time points, with week accounting for approximately 9% of the variance in therapy attitudes. Post hoc comparisons using Tukey’s Honestly Significant Difference indicated that TAI scores at week 4 were significantly lower than at week 8 (*P*=.008) and week 12 (*P*=.047), whereas weeks 8 and 12 did not differ (*P*>.99). Overall, these findings indicate that therapy attitudes increased between weeks 4 and 8 and were maintained through week 12.

**Figure 1.**
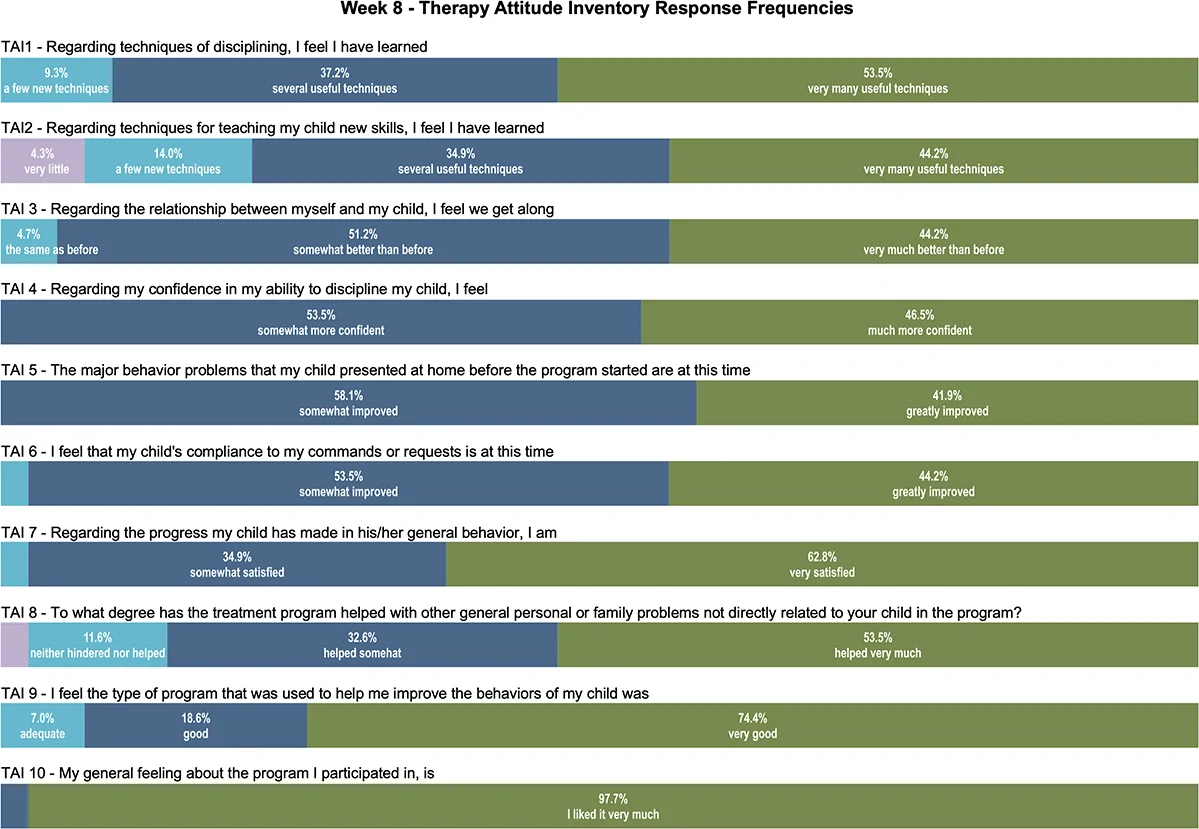
Week 8 Therapy Attitude Inventory (TAI) response frequencies. Note frequencies of responses that were below 4% are not displayed for visual purposes.

At week 8, the items with the highest frequencies of positive responses were #10 (“My general feeling about the program I participated in is”), #9 (“I feel the type of program that was used to help me improve the behaviors of my child was”), and #7 (“Regarding the progress my child has made in his or her general behavior, I am”; Figure 1).

### WAI

**Figure 2.**
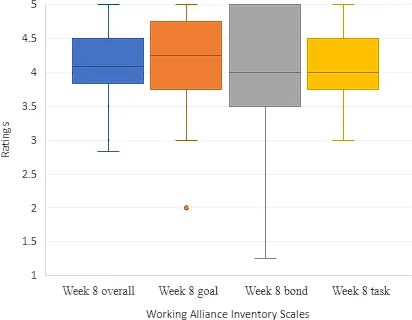
Week 8 Working Alliance Inventory. Note that week 8 is displayed because it has the largest sample size, with most participants completing the entire intervention by then. Extremes represent the range, the box represents the 25th and 75th quartiles, and the bar in the box represents the median. Dots represent outliers.

For the ANOVA comparing WAI scores across the weeks, Levene’s tests indicated that the assumption of homogeneity of variance was met for all analyses. Mean total scores were similar at week 4 (mean 4.10, SD 0.63), week 8 (mean 4.10, SD 0.55), and week 12 (mean 4.04, SD 0.63), and the ANOVA was not significant (*F*_2, 114_=0.182; *P*=.83). Likewise, no significant differences were found for the Goal subscale (wk 4: mean 4.09, SD 0.92; wk 8: mean 4.19, SD 0.66; wk 12: mean 4.13, SD 0.70; *F*_2, 114_=0.182; *P*=.84), the Bond subscale (wk 4: mean 4.03, SD 1.07; wk 8: mean 3.92, SD 1.06; wk 12: mean 4.06, SD 1.01; *F*_2, 114_=0.162; *P*=.85), and the Task subscale (wk 4: mean 3.93, SD 0.74; wk 8: mean 4.06, SD 0.52; wk 12: mean 3.94, SD 0.70; *F*_2, 114_=0.570; *P*=.57; Figure 2).

Orange is week 8 overall, yellow is week 8 agreement with goals, blue is positive bond, and green is agreement with tasks.

## Discussion

### Principal Findings

This study examined caregiver engagement, satisfaction, and therapeutic alliance in a hybrid PMT group program supported by Pat. Consistent with the study aims, caregivers demonstrated high levels of engagement with Pat, reported strong satisfaction across multiple time points, reported progress in their major challenges, and formed a strong therapeutic alliance with Pat. The findings support the feasibility and acceptability of integrating an AI CA into a therapist-led PMT program. These findings should be interpreted in the context of a descriptive study design and are intended to inform the feasibility and acceptability of this hybrid intervention model. Caregivers showed substantial engagement with Pat across the intervention. By week 4, participants had already exchanged an average of 383.09 (SD 185.64) messages with the platform, and message volume was even higher among those assessed later in treatment, with averages of 605.00 (SD 268.61) messages in the 12-week cohorts (who had fewer modules) and 726.47 (SD 405.41) messages in the 8-week cohorts (who had more modules available). This pattern suggests that caregivers interacted with Pat extensively throughout the program. Engagement was also reflected in module completion. On average, caregivers completed 5.41 (SD 1.93) modules by week 4 and 8.67 (SD 3.39) by week 8, indicating that most participants progressed through a substantial portion of the intervention content. Additionally, the optional Q&A use showed a similar pattern, rising from 5.17 (SD 4.75) interactions at week 4 to 10.49 (SD 9.18) at week 8, suggesting that parents completed at least 1 module and 1 in-the-moment support chat per week. Additionally, engagement with Pat was frequent, with an average of 10.00 (SD 5.15) days at week 4, 17.42 (SD 9.26) days at week 8, and 18.90 (SD 9.53) days at week 12. Taken together, these findings suggest that caregivers engaged with structured learning modules and returned to Pat for in-the-moment support to apply parenting skills in real-life situations.

The combination of high message volume, substantial module completion, and repeated Q&A use is consistent with, and notably higher compared with, the existing literature on CAs for parenting support. Engagement in digital interventions is often reflected in sustained participation over time, repeated interactions, and continued use beyond the required intervention activities. In this study, caregivers completed the assigned modules as well as initiated optional in-the-moment support chats, reflecting an engagement level beyond the minimum program requirement. More specifically, to our knowledge, the number of messages exchanged at week 8 (mean 726.47, SD 405.41 messages) is the highest reported in any AI study on mental health. The most comparable study using generative AI for parenting was by Rivera-Cepeda et al [18], which found that four sessions combining therapist support and Pat resulted in an average of 376 messages exchanged. Other parenting studies have generally used earlier conversational technologies and/or shorter interventions and have reported substantially lower levels of interaction [14,16]. A study by Heinz et al [23] using generative AI for depression, anxiety, and body shape concerns with college students yielded an average of 260 messages (sent by users only) at week 8. Thus, the engagement level seen in this study is notably high.

The high level of engagement can be explained by the hybrid nature of the intervention. Pat was integrated into a therapist-supported model in which caregivers received professional guidance from the therapist, personalized reminders from the AI, structured modules, and opportunities to practice skills through role plays with Pat. During the live meetings, the professional assigned caregivers a specific module to be completed with Pat, and at the following meetings, those modules were discussed. Unlike traditional digital parenting programs that primarily deliver educational content, Pat used conversational AI to teach the foundational aspects of parenting skills, promote skills practice through role plays and reminders, and provide immediate support tailored to caregivers’ ongoing parenting challenges. Interestingly, caregivers completed 1 module a week on average and did 1 “in-the-moment” support chat every week. These chats were spontaneously initiated by the parents; therefore, engagement extended beyond the assigned activities. Notably, instead of having just 1 group session a week, parents had 3 support sessions total: the live session, the module with Pat, and the in-the-moment support with Pat, meaning Pat could provide considerably more support to parents.

Rivera-Cepeda et al [19] showed that caregivers experienced Pat as useful and supportive, and they attributed more progress to their chats with Pat than to their participation in the group. It is plausible that when caregivers experience the intervention as helpful in managing real-life parenting challenges, this reinforces continued engagement with the platform. Overall, these findings suggest that generative AI can support considerable caregiver engagement and that such engagement may be amplified when the technology is embedded within a human-supported model of care.

Caregivers reported high levels of satisfaction with the intervention, as reflected in an overall NPS of 76.92, which is considered excellent and indicative of a strong likelihood of recommending the program to others. NPS scores remained consistently high across time points, with values of 77.8 at week 4, 76.7 at week 8, and 75 at week 12. This pattern suggests that positive perceptions of the intervention were established early and maintained throughout the program and did not decline with continued use of the CA. One possible explanation is that early interactions with Pat facilitated the formation of positive initial impressions, as caregivers were able to immediately access guidance and support during real-life parenting challenges, allowing them to experience the relevance and usefulness of the intervention early on. This was reinforced as caregivers continued to engage with the platform and apply parenting strategies in real-world contexts. Rivera-Cepeda et al [19] reported that a qualitative analysis of caregivers’ experiences showed that they valued constant availability and in-the-moment support as some of the aspects most valued by the parents.

These findings are consistent with prior research on parenting CAs, which has demonstrated high levels of satisfaction and acceptability [14]. Similarly, systematic reviews have found high acceptability and retention rates for CA interventions in parenting and mental health contexts for children and adults [15]. The strong NPS observed in this study aligns with this growing body of literature and further supports the acceptability of AI-augmented parenting programs. Importantly, much of the existing literature assesses satisfaction at a single postintervention time point [15]. Taken together, these findings suggest that an approach that combines human therapists who encourage the use of the AI can sustain parents’ satisfaction across extended engagement, which is critical for the delivery of the parenting skills that Pat taught the parents. Although the acquisition of parenting skills was not measured, this represents an important direction for future research.

Beyond their high willingness to recommend Pat, caregivers reported high levels of satisfaction with the intervention, as measured by the TAI, with mean scores increasing from week 4 to weeks 8 and 12. Overall, the responses from the TAI showed that caregivers reported positive impressions of the program, reported improvements in their children’s behavior, gained confidence, learned practical parenting techniques, and experienced improvements in their relationships with their children. These patterns are consistent with emerging evidence suggesting that perceived therapeutic alliance is associated with engagement and clinical outcomes in digital interventions [24]. Together, these findings indicate that alliance ratings remained stable across the weeks of administration, with consistently strong agreement on therapeutic goals, tasks, and relational bonds.

As caregivers gained experience applying the strategies introduced in the intervention, their confidence and perceived effectiveness may have increased, contributing to more favorable evaluations in later weeks. These findings are consistent with prior research on parenting interventions, which have demonstrated high levels of satisfaction and perceived improvement in both caregiver confidence and child behavior outcomes [22,25]. Previous studies have reported similarly high TAI scores, with caregivers indicating meaningful improvements in parenting skills and child behavior alongside strong satisfaction with the intervention process [25]. However, most prior research has assessed satisfaction at a single time point, limiting the understanding of how caregiver perceptions evolve across treatment. The present study examined satisfaction across treatment, demonstrating that positive perceptions increased across multiple time points. These findings suggest that caregivers responded positively to the intervention and perceived meaningful changes in both their parenting practices and their children’s behavior over time.

Therapeutic alliance ratings with Pat remained consistently high across the intervention, with mean scores of 4.10 (SD 0.63) at week 4, 4.10 (SD 0.55) at week 8, and 4.04 (SD 0.63) at week 12, and no significant differences were observed across time. This pattern suggests that caregivers were able to establish a strong working alliance with the CA early in the intervention and maintain it throughout the program. The stability of these scores indicates that continued interaction with the AI did not diminish the perceived relational bond, agreement on goals, or collaboration on tasks. Overall, the therapeutic alliance scores reported in this study are relatively high compared with both CA and human-delivered interventions [21,26]. Prior research examining alliance with digital CAs has reported lower scores, including means of 3.75 for Wysa [27] and 3.36 for Woebot [26]. Other studies have also demonstrated the formation of therapeutic alliances using alternative measures, with reported scores that range approximately between 4.22 and 4.49 on the Working Alliance Questionnaire [28]. Alliance scores in this study also compare favorably with those reported in some human-delivered psychotherapy contexts, such as the approximately 3.8 reported by Munder et al [21]. The therapeutic alliance scores observed with Pat suggest that caregivers may have experienced a strong sense of connection, collaboration, and support when interacting with Pat. One possible explanation for these findings is that Pat provided caregivers with immediate access to support, opportunities to practice parenting skills, personalized guidance, and assistance applying PMT strategies between sessions. These features may have contributed to perceptions of collaboration, support, and shared goals, which are central components of a therapeutic alliance. It is possible that the high scores reported about Pat are influenced by the hybrid approach that included a human therapist. As such, the alliance formed with Pat should not be interpreted as equivalent to an alliance in individual psychotherapy. Rather, these findings suggest that when humans and AI are combined, this can result in a stronger therapeutic alliance than when each component is isolated. Within the context of a hybrid PMT program, Pat appears to function as more than a supplementary tool; it serves as a relational and collaborative component that supports caregivers in applying parenting strategies in real-world contexts.

### Practical and Clinical Implications

The findings of this preliminary study have several implications for clinicians, program developers, and health systems. For clinicians, hybrid models that combine live PMT through video conferencing with AI support can enhance scalability and continuity of care, allowing families to receive personalized guidance from their homes and receive support between sessions without increasing therapist workload. AI tools like Pat can function as engagement boosters (ie, reminding, reinforcing, and tracking caregivers’ use of skills). While this study did not analyze engagement, other studies on group interventions with adults with depression have shown that AI could enhance engagement with the treatment and improve outcomes [29]. For developers, including accessible resources (such as apps) and voice features to avoid typing will be essential to ensure smoother engagement with the CAs. Finally, for health systems, integrating AI-supported interventions could lead to greater accessibility and cost efficiency, reducing logistical barriers such as time, transportation, and provider shortages that often limit families’ participation in evidence-based parenting programs.

### Limitations and Future Directions

Several limitations should be considered when interpreting these findings. This study was conducted as a real-world implementation without a control group, which limits the ability to draw causal conclusions about the effects of the intervention. Because the design did not include a comparison condition, it is not possible to isolate the specific contribution of the AI CA relative to the therapist-led group or the combined hybrid model. Conclusions regarding the specific effects of Pat should be interpreted cautiously, as engagement, satisfaction, and alliance ratings may reflect the combined influence of the therapist and the AI-supported component. The findings of these analyses should be interpreted as evidence of feasibility, engagement, and acceptability rather than efficacy.

The analysis relied primarily on self-report measures, which may be influenced by social desirability, recall bias, or participant expectations. However, these self-report assessments are commonly used in psychotherapy and digital intervention research, particularly when evaluating subjective experiences such as satisfaction and alliance. Nonetheless, the absence of objective child-level or family-level outcome measures limits the ability to determine whether perceived improvements translated into measurable changes in child behavior or family functioning. Additionally, the analyses are based on parents who chose to complete the surveys, which biases the sample.

The sample consisted largely of highly educated, digitally literate caregivers with consistent internet access, which may limit the generalizability of the findings to more diverse or underserved populations. Additionally, participants who remained engaged in the study and completed assessments may differ systematically from those who did not, introducing potential attrition bias and limiting insight into the experiences of less engaged users.

The intervention evolved over the course of the study, with differences in program duration and the availability of optional modules across cohorts. These variations may have influenced engagement patterns and user experiences, making it challenging to determine whether observed differences were due to the intervention structure or participant behavior. Due to the hybrid format of the intervention, which included therapist-led group sessions alongside interactions with the AI agent, perceptions of satisfaction and therapeutic alliance with Pat may have been shaped by the broader treatment context. As such, it is not possible to determine the extent to which the observed alliance and satisfaction are attributable specifically to the AI agent versus the combined human-AI model.

Future research should address these limitations by incorporating randomized controlled designs that allow for direct comparisons between intervention conditions. In particular, studies comparing group PMT with and without the inclusion of Pat would help clarify the unique contribution of Pat to the outcomes. Additionally, future studies should expand beyond experimental comparisons and examine the relationships and mechanisms underlying these outcomes. While the present analysis provides evidence of feasibility, engagement, and acceptability, it does not examine how these variables interact or contribute to one another. Understanding these relationships will be crucial for identifying the mechanisms through which AI-supported interventions influence caregiver experiences and outcomes.

Although the inclusion of caregivers from Latin American countries represents a strength in terms of cultural context, future research should aim to include more socioeconomically and educationally diverse samples, particularly those with varying levels of digital literacy and access to resources, in order to better assess the generalizability and scalability of Pat. Finally, because of the hybrid format, ratings of engagement, satisfaction, and therapeutic alliance may reflect the combined influence of the therapist-led and AI-supported components rather than the AI component alone and therefore should be interpreted cautiously.

### Conclusions

Caregivers reported high engagement, satisfaction, and therapeutic alliance with a hybrid PMT program combining a human therapist and an AI CA. Caregivers interacted extensively with the platform, completing assigned modules and using optional in-the-moment support chats, suggesting that the intervention was used consistently and integrated into daily parenting practices. Satisfaction remained high across time, and caregivers reported positive therapy attitudes, indicating that the program was perceived as helpful, relevant, and worth recommending to other caregivers. Therapeutic alliance was also strong and stable across the intervention, which suggests that caregivers were able to establish a meaningful working relationship with Pat. A notable finding was the value of the in-the-moment support, which allowed caregivers to apply parenting strategies directly to real-life situations. These findings suggest that integrating AI with a human therapist is feasible and acceptable within a group PMT program and may provide additional support between scheduled sessions. Parents were open to this hybrid model and utilized the support provided by the AI in their everyday lives.

## Abbreviations

CA: conversational agent
NPS: Net Promoter Score
PMT: parent management training
TAI: Therapy Attitude Inventory
WAI: Working Alliance Inventory
WAI-SR: Working Alliance Inventory–Short Revised
